# Linguistic perspective-taking in verbal communication with LLMs: evidence of an egocentric bias

**DOI:** 10.3389/fpsyg.2026.1894763

**Published:** 2026-08-07

**Authors:** Lea Marie Petrasch, Regina Jucks

**Affiliations:** Faculty of Psychology and Sports Science, University of Münster, Münster, Germany

**Keywords:** chatbots, higher education, illusory transparency of intention, large language models, perspective-taking

## Abstract

Since the expansion of generative AI’s functions, chatbots such as Open AI’s Chat-GPT appear as more than tools. They can be perceived as communicative partners, shaping exchanges with high variability and linguistic competence. Current versions, such as Chat-GPT 5.5, even provide spoken language using different verbal styles and vocabulary, intonation and prosody. Investigating this new context of communication, we question whether people expect chatbots to detect non-literal meanings. Extending the *illusory transparency of intention* research paradigm introduced by Keysar, information was presented through scenarios of interactions with the Chat-GPT voice function that disambiguated the presented verbal information as either non-literal (sarcastic) or literal (sincere) in an within-subject experimental design (*N* = 327). Scenarios were placed within the higher education context. Results reveal that participants did not only falsely expect the chatbot to detect a non-literal meaning, but they also even expected the chatbot to reply accordingly. Implications for higher education contexts are discussed.

## Perspective-taking in Human-AI interactions

“In many ways, the magic of AIs is that they can convince us, even knowing better, that we are in some way talking to another mind” (Mollik, 2024, p. 211). For the first time in history, we as humans have created something, a machine, that we can talk to and get reasonable answers from. These Large Language Models (LLMs) are no longer just mediums we communicate through, but they are designed to take on the role as communicators (Guzman and Lewis, 2020). One well documented bias in human-human communication is the illusory transparency of intention (Keysar, 1994; Lau et al., 2022): people apply their privileged knowledge about the intention behind an utterance to the addressee. In Keysar’s (1994) seminal study, participants assumed that the protagonist Mark successfully transported his dislike for a restaurant (as a sarcastic reply) to the (unknowledgeable) coworker June (Gerrig et al., 2000a, 2000b; Keysar, 1994, 2000).

So far, perspective-taking and the egocentric biases that occur have not been studied systematically in Human-AI interactions. In this study we used an experimental design from Keysar (1994) and transformed it to the context of Human-Machine Communication (HMC) (Guzman, 2018) to answer the question: Do people (illegitimately) expect LLMs to detect the conveyed meaning behind their messages?

Firstly, we will discuss previous work on perspective-taking and the impact of privileged information, especially in spoken communication. From there we move on to the communication with machines, specifically LLMs. Following this, we formulate our research questions and hypotheses.

## The overestimation of shared understanding

There have been many studies about people’s overestimation of shared understanding for example in conveying an intended meaning in ambiguous sentences (Keysar and Henly, 2002) or conveying sarcasm via e-mail (Kruger et al., 2005). This overestimation stems from an egocentric bias, namely people taking their own perception as their reference point for information processing (Oeberst and Imhoff, 2023). One of such overestimations of shared understanding is the illusory transparency of intention (Keysar, 1994). In four experiments participants were introduced to different scenarios of two co-workers, friends, or similar having a conversation. Each scenario ended with an ambiguous statement of the speaker. Participants then judged whether the addressee perceived sarcasm. In cases with negative privileged information the intention of the statement could be seen as sarcastic, in cases with positive privileged information the statement did not seem sarcastic. The results showed that the knowledge of either negative or positive privileged information had significant influences on how participants thought the person in the scenario would interpret the ambiguous sentence. Keysar concluded, that having “information about the speaker’s intention made participants more likely to believe that the intention would be perceived by the addressee” (p.199).

Another instance is the extreme illusion of understanding (Lau et al., 2022) where listeners and speakers (the sample was randomly assigned to the roles) both showed an overestimation of their understanding. To test this, speakers had to record an ambiguous phrase with one assigned meaning (out of four possible). They then rated whether the listener would identify the meaning (estimated accuracy) and how confident they were. Listeners listened to the recording and identified the meaning (actual accuracy) and also rated how confident they were. The results showed that speakers and listeners overrated the success of their communication. In a second round speakers and listeners did not share the same language (American and Chinese). Nevertheless, the effect of overestimation was still significant.

## The coordination of perspectives

How can the overestimations illustrated above be explained? We will briefly outline the theoretical background. “Every joint activity requires coordination among its participants” (p. 35) (Clark, 2009) for example playing a four-handed piano piece – a principle that likewise applies to language use. These so-called social factors of language use are still an active research field today (Achimova et al., 2025; Barr and Keysar, 2006). When speaking or writing to each other, we have to coordinate our own and our interlocutor’s shared knowledge, beliefs and suppositions which is referred to as assessing the common ground (Fussell and Krauss, 1992). In order to do so, we have to infer the other person’s perspective (perspective-taking). The process of perspective-taking is essential in successful communication (Nickerson, 1999) and has been studied in developmental psychology as well as in adults (Dumontheil et al., 2010; Epley et al., 2004a, 2004b). However, like most human judgements, perspective-taking is susceptible to bias, in this case egocentrism (Gilovich and Savitsky, 1999). This becomes evident when privileged information is introduced, as illustrated by the two studies in the paragraph before. Studies manipulating privileged information, that is information only one interlocutor knows, show the tendencies of people imputing their knowledge onto others (Nickerson, 1999). Royzman et al. (2003) described this “perspective-taking failure” as the “difficulty to set aside own privileged knowledge in predicting the knowledge state of another (nonprivileged) person” (p. 61).

Within this study we especially want to focus on verbal or spoken communication. In his experiments, Keysar (1994) manipulated two elements, privileged information and the modality of the utterance, which was either spoken or written. He argued that in the spoken modality participants might expect the intonation of the speaker to transport the intended meaning. However, in the written modality they cannot expect any differential cuing between the two conditions of privileged information. Therefore, any effect of privileged information in the written modality would speak for an illusory transparency of intention. Indeed, he found the effect of privileged information in both conditions. There was no difference for the perceived sarcasm of the addressee whether the utterance was spoken or written. Still, we would like to point out that the difference between the two modalities was actually only within the description of the scenario, which were always written. Participants did not actually experience a spoken version. This study builds on precisely this point.

Another study investigating speakers’ overestimation of their effectiveness (Keysar and Henly, 2002) found similar effects to Lau et al. (2022), where speakers’ overestimated their success of communicating a specific intention behind ambiguous sentences. Furthermore, in a second experiment they added overhearers, participants who shared the same information as the speakers but only heard the spoken sentences (did not speak themselves). Interestingly even though they had access to the same knowledge as speakers, they did not overestimate listeners understanding. The authors therefore conclude, that knowledge of the intended meaning alone cannot explain speakers’ overestimation.

Along similar lines, Kruger et al. (2005) studied overestimations in communication via e-mail. In their fourth experiment they had participants read the e-mail out loud after they finished typing either with the same (intended) phenomenology or an opposite phenomenology. For the first group the results showed the same overestimation (discrepancy between estimated and actual accuracy) as the experiments before. Whereas for the second group with an opposite phenomenology the effect disappeared completely. When participants vocalized their statements inconsistent with the intended meaning, estimated and actual accuracy were on the same level and the overconfidence disappeared.

In summary, studies on spoken communication do not show a clear picture. There have been findings of overestimations in speaking and listening, in other studies no effects can be found. Comprehension of spoken references remains an active research field (Barr and Keysar, 2006) and will be further addressed through this study.

## Human-machine communication

The Computers Are Social Actors (CASA) paradigm (Nass et al., 1994) describes the phenomenon of people applying social rules to their interactions with computers. They therefore argue that the “human-computer relationship is fundamentally social” (p.77). Since its first publication, the paradigm has been extended, changing the assumption of mindless application of human-human social scripts to humans applying human-media social scripts in such interactions (Gambino et al., 2020). Most recently, it was argued that the CASA effect is short-lived and only applies to emerging technologies (Heyselaar, 2023). In their direct replication of the experiment, they could not find a CASA effect for desktop computers and attributed this to the societal change and the advancement of technology since then. Nevertheless, as chatbots and LLMs are still emerging, we expect that the CASA paradigm applies to them. Furthermore, we would suggest that the social cues they transmit are more advanced compared to the social cues a desktop computer could transmit (see also Guzman and Lewis, 2020).

Moreover, Human–AI communication is shaped by social perceptions and interpersonal communication norms. Students evaluate conversational systems not only according to their functionality but also in terms of politeness, warmth, benevolence, and trustworthiness (Brummernhenrich et al., 2025; Jucks et al., 2018; Plagge and Jucks, 2025). Anthropomorphic communication cues can increase a chatbot’s perceived social presence and thereby influence users’ trust in and responses to the system (Konya-Baumbach et al., 2023). However, the effects of human-like attributions are not uniform: attributing intelligence to a large language model can promote reliance on its advice, whereas attributing emotional experience can reduce it (Colombatto et al., 2025). Human–AI communication can therefore be understood as a socially situated interaction in which communicative cues and users’ interpretations of the system jointly shape trust and engagement. If someone, or in this case something, seemingly speaks our language, we as humans are likely to attribute communicative intent to this linguistic signal (Bender and Koller, 2020). With high quality interactions, LLMs are specifically designed to make us feel as if we were interacting with a person and this has been amplified even more by adding the ability to speak (voice function) (Mollik, 2024; OpenAI, 2026). Already Weizenbaum (1976) described the phenomenon that after a short exposure to a simple computer program, people anthropomorphized the system and were emotionally attached to it (see also Epley and Waytz, 2010; Holtgraves et al., 2007).

Therefore, this study focuses on the voice function of LLMs. Voice-based and AI-infused agents have become increasingly popular and integrated into our everyday lives, like Apple’s Siri or Google’s Alexa, but still voice-based interactions remain understudied (Seaborn et al., 2022). Voice can be seen as a fundamental feature of social interaction (Seaborn et al., 2022) and for the first time it does not only involve humans (Gunkel, 2012; Guzman, 2018). Detailed, personal conversations with generative AI are no longer a futuristic fantasy, but part of our reality, and are increasingly shaping our society (Hepp et al., 2023) and cognition (Shanmugasundaram and Tamilarasu, 2023). To our knowledge this has not been studied extensively in communication with LLMs.

## Research question

With this investigation we connect the field of cognitive psychology, namely perspective-taking to the social aspect of communication with LLMs. Specifically, we will explore whether the illusory transparency of intention applies to verbal human-AI communication. Do errors in perspective-taking caused by an egocentric bias that arise in human-human communication apply? We hereby contribute to the research field of voice-based interactions with LLMs which to our knowledge has not been studied systematically.

## Present study

To examine our research question, we implemented a 1 × 2 within-subject design based on Keysar (1994), manipulating privileged information. Depending on the valence of privileged information provided, the scenarios could be interpreted with two different conveyed meanings: the last statement of each scenario could either be interpreted as literal (sincere) or as non-literal (sarcastic) (Keysar, 2000). Our dependent variables were *participants’ judgements of chatbots’ processing of sarcasm*, indicating whether they believed the chatbot recognized sarcasm, as well as the *prediction of a future action/reaction*. To see if the manipulation of privileged information worked as intended, we also measured *participants’ own perception of sarcasm*.

### Hypotheses

Assuming that human-computer interactions follow similar social scripts, we would expect individuals to exhibit a comparable egocentric bias as observed in human-human communication. Following Keysar’s (1994) study design, we manipulated privileged information to examine whether the illusory transparency of intention applies. Based on this, we propose two hypotheses:

*Hypothesis 1*: Privileged information significantly influences *participants’ judgements of chatbots’ processing of sarcasm*. With privileged information suggesting a non-literal meaning of the last utterance, participants assume the chatbot would also perceive a non-literal (sarcastic) meaning; with privileged information suggesting a literal meaning, they assume the chatbot would also perceive a literal (sincere) meaning.

*Hypothesis 2*: Privileged information significantly influences the *prediction of a future action/reaction*. With privileged information suggesting a non-literal meaning, participants are more likely to expect a sarcasm-congruent response; with privileged information suggesting a literal meaning, they are more likely to expect a sarcasm-incongruent response.

*Participants’ own perception of sarcasm* will serve as a manipulation check. Scenarios with privileged information suggesting a non-literal meaning should receive higher values (i.e., on a scale toward sarcasm); the opposite is expected for scenarios with privileged information suggesting a literal meaning.

Finally, we want to point out that any differences between our two conditions cannot be attributed to differential cueing, like sarcastic intonation (Keysar, 1994). Since scenarios were narrated by the Chat-GPT voice function the last statement of each scenario was pronounced neutrally independent of a literal or non-literal meaning.

## Methods

### Procedure

This study was preregistered at OSF^1^ prior to data collection, and it was approved by the ethics committee of the Faculty of Psychology and Sports Science at the University of Münster. Data, analysis code, and all materials used in the study are openly accessible through our OSF project under files^2^. Participants were recruited and compensated via the Prolific Academia panel, receiving £3 upon survey completion, in accordance with the panel’s recommendations. To avoid priming effects, the survey introduction provided only general information about the study’s purpose (i.e., general perception of chatbots). Upon completion of the survey, participants were debriefed and informed about the specific research question namely, the attribution of detecting conveyed meaning to chatbots in the context of privileged information and egocentric bias.

### Study design and materials

**Figure 1:**
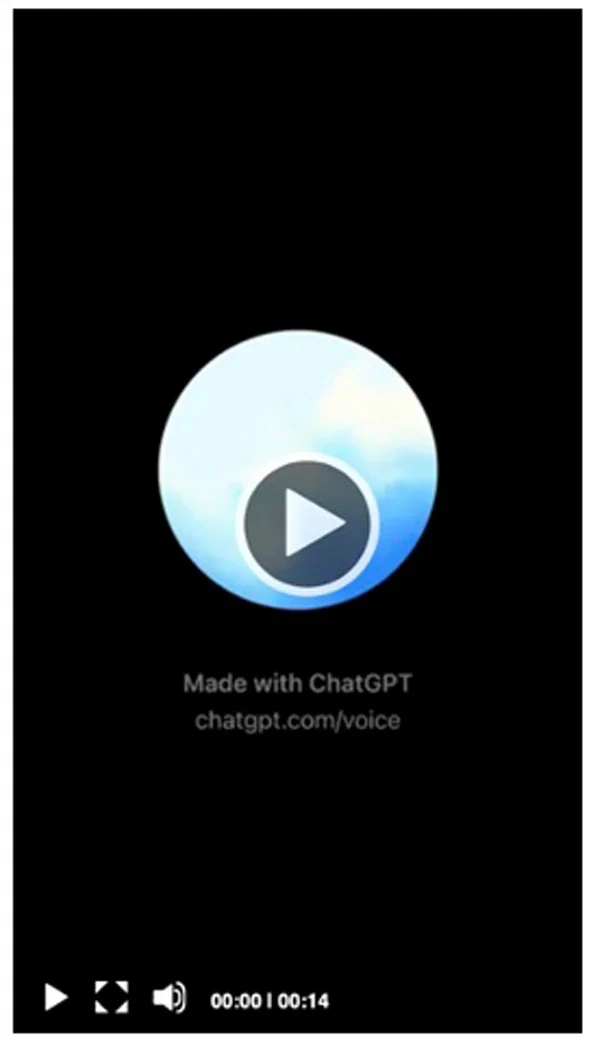
Scenario 6-Hannah asks about the cafeteria’s opening hours Introductory text: It is in the afternoon and Hannah, a college student, has not had lunch yet. She asks the chatbot Frankie how much longer the cafeteria will be open. Transcription of privileged information with literal intention: She’s in luck-the cafeteria is still open for another 30 min. She heads there right away. When she arrives at the cafeteria 10 min later, there are many other students there who are just getting something to eat. She received the right information and goes straight to the food line. She replies, “Thanks for sending me to the cafeteria.” Transcription of privileged information with non-literal intention: She’s in luck-the cafeteria is still open for another 30 min. She heads there right away. When she arrives at the cafeteria 10 min later, she finds the doors locked. Not only has the food line closed, but the cafeteria has already shut down completely. She was given the wrong information and is still hungry. She replies, “Thanks for sending me to the cafeteria”.

To test our hypotheses, we conducted an online experiment employing a 1 × 2 within-subject design, in which privileged information was manipulated as either suggesting a literal or non-literal meaning. Following the research design introduced by Keysar (1994), we created scenarios of student-chatbot interactions, where the student’s final remark could be interpreted as either literal (sincere) or non-literal (sarcastic). As each participant was intended to view only one version of each scenario, two complementary scenario sets (Set 1 and Set 2) were constructed. First, we randomized the assignment to the literal or non-literal condition for each scenario, ensuring that each set ultimately contained three literal and three non-literal scenarios. Afterward, the order of the scenarios within each set was randomized. Lastly, participants were randomly assigned to set 1 or set 2. The scenarios described common situations in university students’ lives (e.g., canteen opening hours, exam stress, etc.). Each scenario began with a brief introductory text that was identical across conditions. Privileged information was embedded within student-chatbot interactions, which were illustrated through screenshot recordings of the ChatGPT voice function (see Figure 1 for an example). We used the voice “Maple” which is described as cheerful and candid across all scenarios. All study materials were presented in German.

### Measures

After providing informed consent, participants completed a practice example (similar to Keysar, 1994). In the first round, they were presented with six scenarios (three per condition) along with five filler scenarios. The fillers followed the same structure as the scenarios but did not imply sarcasm and were inserted between scenarios. Following each scenario (and also filler), participants indicated the *chatbot’s processing of sarcasm* (e.g., Did Frankie (the chatbot) take Hannah’s comment as sarcastic?) using a *Yes-Maybe-No* scale (similar to Keysar, 1994). This measure assessed whether participants (erroneously) incorporated privileged information. To prevent potential “personality” effects, the names of both chatbots and students were varied across scenarios. Chatbot names were gender-neutral, whereas student names were common German names. In a second round, the scenarios were presented again, this time without fillers in between. Participants were asked about their *own perception of sarcasm* (e.g., Did you think Hannah was being sarcastic?), again using a *Yes-Maybe-No* scale. Additionally, we wanted to know what participants thought the chatbot would probably answer. For this we used the open answers of one of our former studies (Petrasch & Jucks, 2025) and formulated one sarcasm-congruent and -incongruent answer for each scenario between which they had to choose. For example, for the cafeteria scenario ending in the ambiguous utterance “Thanks for sending me to the cafeteria.” the first option was “I’m glad I could help you. Enjoy your meal in the cafeteria!” (sarcasm-incongruent), and the second option was “Sorry for the incorrect information! Let me know if there is anything else I can do to help.” (sarcasm-congruent). Lastly, we collected demographic information concerning age, gender, and language experience in German. Concerning chatbots we asked about former experience with one item (“How much experience do you have using generative AI, such as for example Chat-GPT, Gemini or Copilot?”) on a five point Likert-scale (No experience, little experience, moderate experience, extensive experience, and very extensive experience) and frequency of use with one item (“How often do you use generative AI, such as for example Chat-GPT, Gemini, or Copilot?”) on a five point Likert-scale (Never, Rarely (less than once a month), Relatively often (Multiple times per month), Often (Multiple times per week), Very Often (Daily)). We used the same two items to measure former experience and frequency of use of the voice function.

### Sample

For our target sample size, we ran an *a priori* analysis using G*Power (Faul et al., 2007). For a two-tailed *t*-test comparing two dependent means, an alpha error of 0.05, power of 0.95, and an expected small effect (*d* = 0.2), the calculated sample size was 327 people.

Our inclusion criteria required that participants were located in the DACH region, their first language had to be German, and they had to be currently enrolled at university or college. In total, *N* = 331 people completed the survey who met the inclusion criteria. We followed Meade and Craig’s (2012) recommendation to ask for consent at the beginning and the end of our survey. Furthermore, to ensure high-quality data we included attention and comprehension checks throughout the study (Muszyński, 2023; Peer et al., 2022). Our first comprehension check followed after the second filler and asked participants in a single-choice question with four options about the content of the previous scenario. Also, we included an attention check in form of a “fake scenario” where the introductory text to the scenario looked similar to all other scenarios, but the video did not contain the continuation of the scenario but an instructed response (Ejelöv and Luke, 2020). Lastly, in the second round of scenarios we included one comprehension check asking participants about the content of the previous scenario with an open question. We excluded two participants who failed the first two checks and two participants who did not play the videos. Our final sample included *N* = 327 participants.

**Figure 2:**
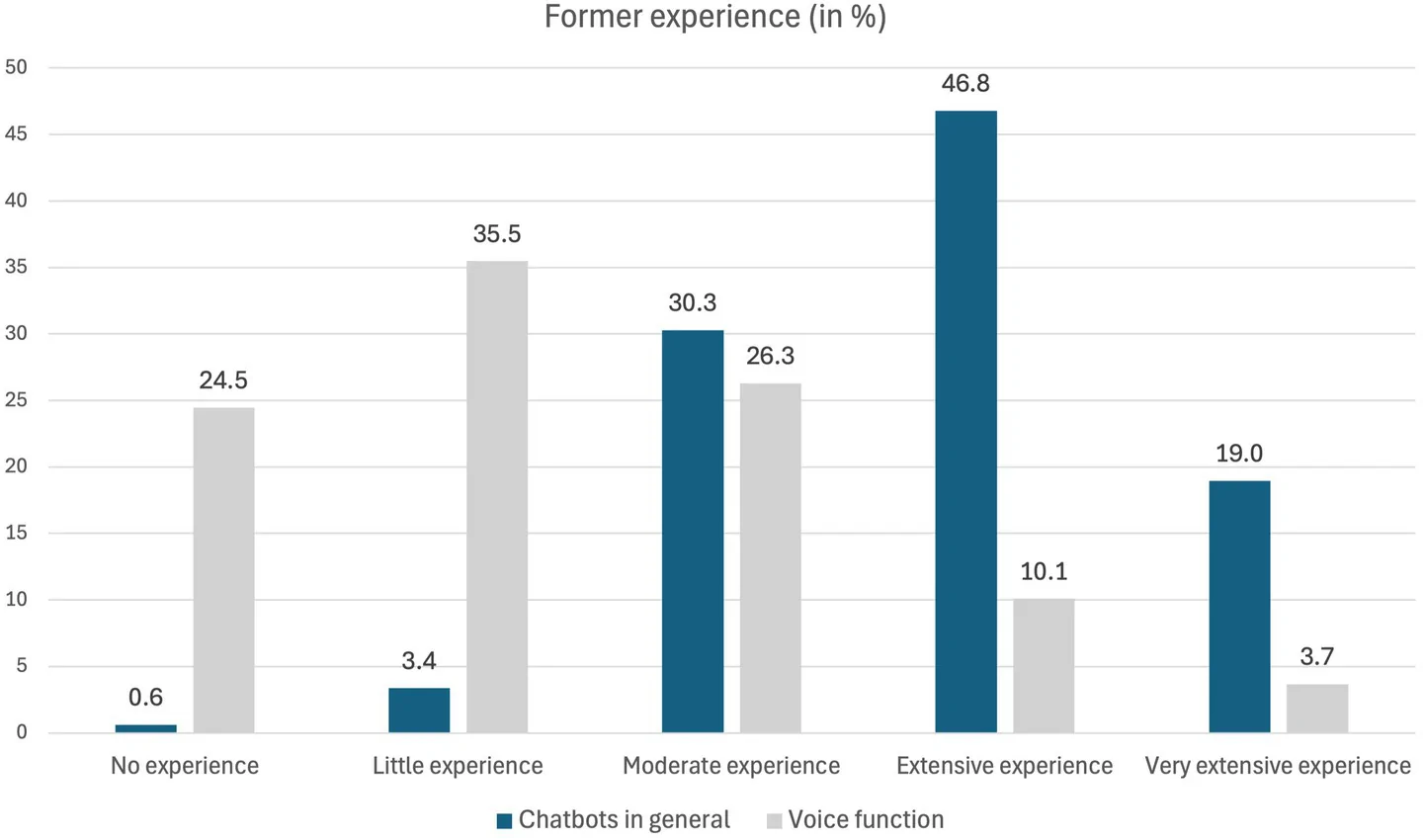
Former experience with chatbots and their voice functions. *N* = 327.

**Figure 3:**
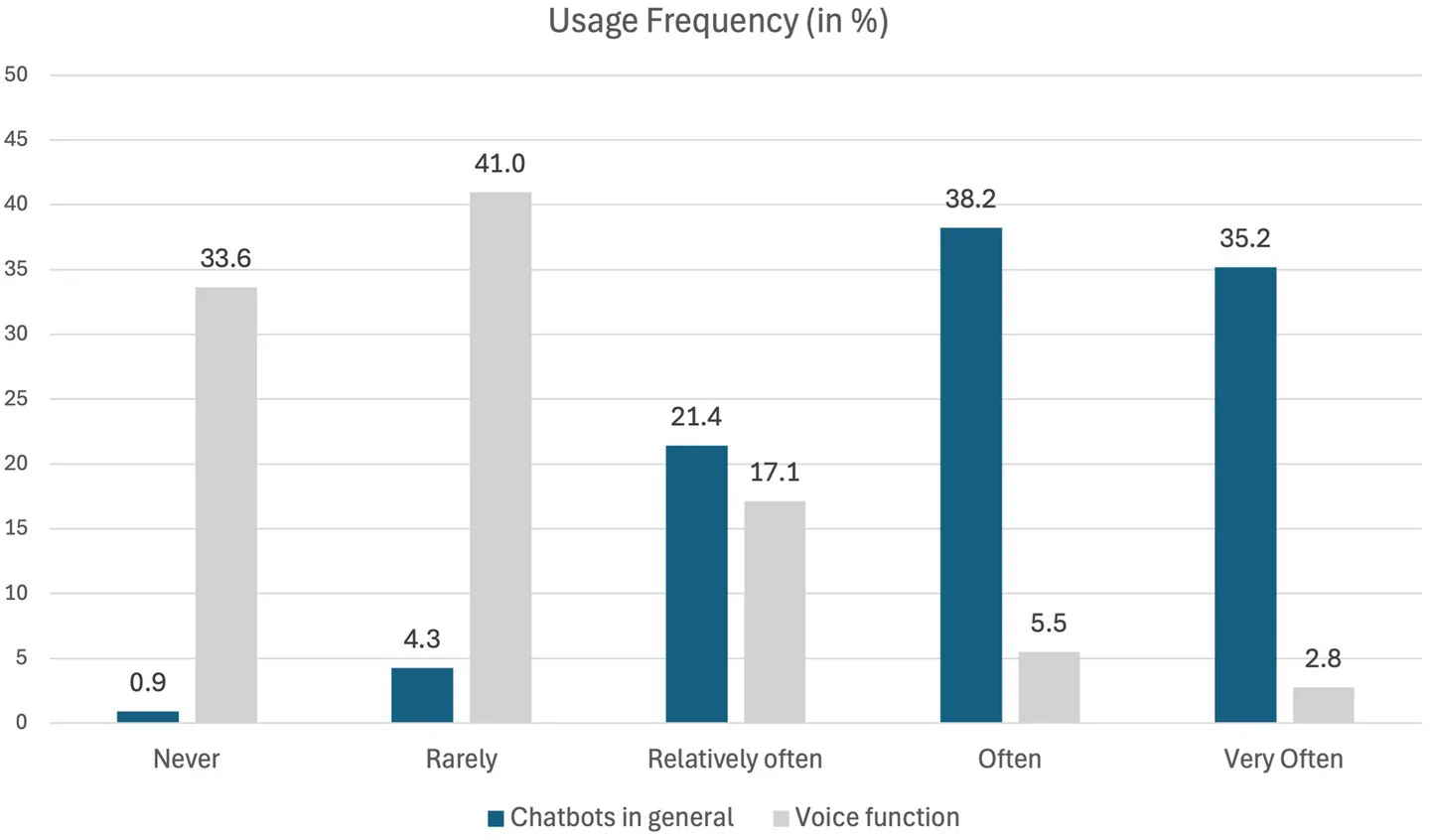
Usage frequency of chatbots and their voice functions. *N* = 327.

The age of participants ranged from 18 to 42 years (*M* = 24.66, *SD* = 4.59). With regard to gender, 70% of participants identified as male, 28.7% as female, 0% as diverse, and 1.2% preferred not to provide this information. Only two participants were not German native speakers, with four and 18 years of language experience. Former experience with chatbots and their voice functions (Figure 2) and frequency of using both (Figure 3) are displayed below.

In total, 65.8% of our sample had extensive or very extensive experience with chatbots, whereas only 13.8% where in those two categories for the voice function. A very similar pattern emerged for the usage frequency: 73.4% were frequent or very frequent users of LLMs, only 8.3%, respectively, for the voice function.

## Results

Data were analyzed using IBM SPSS statistics (IBM Corp, 2023). Strictly following Keysar (1994), analyses were conducted separately for the dependent variables, namely the *participants’ own perceptions of sarcasm*, their *judgements of chatbots’ processing of sarcasm*, and their *prediction of a future action/reaction*. To assess statistical differences between conditions, we employed two complementary approaches. Consistent with the methodology of the original study (Keysar, 1994), we computed combined scores and compared them across conditions. Second, as all three dependent variables were categorical or dichotomous, ordinal mixed models were employed to accommodate the structure of the data.

*Participants’ own perceptions of sarcasm.* Table 1 presents the distribution of responses across the individual scenarios. Participants more often perceived sarcasm in messages that followed events implying a sarcastic interpretation (privileged information suggesting a non-literal intention). On average, sarcasm (i.e., a “Yes” response) was identified in only 1.63% of cases when it was not suggested. In contrast, when sarcasm was suggested, participants judged it to be present in 82.12% of cases.

**Table 1:** Percentage of answers (rounded) attributing the intention of sarcasm to speakers per scenario.

Scenario	Privileged information					
	Non-literal intention			Literal intention		
	%Yes	%Maybe	%No	%Yes	%Maybe	%No
1 Daniel struggles with exam preparations	90	4	6	4	5	91
2 Daria curates next semester’s timetable	90	3	7	3	5	92
3 Tom registers for his exams	81	7	12	0	4	96
4 Franzi wants to join the basketball team	63	21	16	1	2	97
5 Ben navigates to the library	86	3	11	1	1	98
6 Hannah asks about the canteen’s opening hours	83	9	8	1	2	97

Responses were coded as follows: “Yes” responses were given a value of 1, “No” responses a value of −1, and “Maybe” responses a value of 0. To compute a combined sarcasm score, scores were averaged for each participant across scenarios within the same condition, resulting in one mean score per condition. A significant difference between the two conditions served as a manipulation check, indicating that the manipulation of privileged information affected participants’ own perception of sarcasm. The two conditions were tested against each other in a matched-pairs *t*-test. On average, participants perceived more sarcasm in the non-literal condition (*M* = 0.72, *SD* = 0.53) than in the literal condition (*M* = −0.94, *SD* = 0.19). This difference of 1.66, BCa 95% CI [1.59, 1.72], was significant, *t*(326) = 49.21, *p* < 0.001, corresponding to a Cohen’s *d* = 2.72. According to Cohen (1988), this represents a large effect size. Additionally, a generalized linear mixed model (GLMM) with a multinomial distribution and a cumulative logit link function was fitted, accounting for the ordinal nature of the dependent variable and the repeated-measure design. Condition was entered as a fixed effect, scenario as a fixed control factor, and a random intercept for participants was specified to account for the dependency of repeated measurements within participants. Effects are reported as odds ratios with 95% confidence interval. Condition had a significant effect on participants’ *own perception of sarcasm*, *F*(1,1954) = 840.02, *p* < 0.001. With literal privileged information participants were much less likely to perceive sarcasm (i.e., give a “Yes” answer) than with non-literal privileged information (OR = 0.003, 95% CI [0.002, 0.004], *p* < 0.001). A significant random-intercept variance (Var = 1.15, *p* < 0.001) reflected substantial interindividual differences in the overall tendency to perceive sarcasm.

These findings support the assumption that the scenario versions were perceived as intended: Following privileged information suggesting a non-literal meaning, participants were more likely to interpret the speaker’s comment as sarcastic compared to following privileged information suggesting a literal meaning. This suggests that participants employed privileged information to infer speakers’ (i.e., students’) intentions. However, this conclusion must be drawn with caution, as participants provided these judgements after considering the chatbot’s processing.

*Judgements of chatbots’ processing of sarcasm*. Participants attributed the processing of sarcasm to the chatbot in accordance with privileged information. Whereas 1.42% of respondents selected “Yes” when the event did not imply sarcasm, 35.8% indicated the chatbot would process sarcasm when the event information suggested a sarcastic interpretation. Table 2 presents the percentages of Yes, Maybe and No choices for each scenario.

**Table 2:** Percentage of answers (rounded) attributing the processing of sarcasm to chatbots per scenario.

Scenario	Privileged information					
	Non-literal intention			Literal intention		
	%Yes	%Maybe	%No	%Yes	%Maybe	%No
1 Daniel struggles with exam preparations	35	24	41	1	19	80
2 Daria curates next semester’s timetable	41	27	32	3	16	82
3 Tom registers for his exams	24	24	52	1	5	94
4 Franzi wants to join the basketball team	32	38	30	3	6	92
5 Ben navigates to the library	43	13	44	0	8	92
6 Hannah asks about the canteen’s opening hours	40	30	30	1	9	91

As with participants’ own perceptions, combined sarcasm scores per condition were calculated (Yes = 1, No = −1, and Maybe = 0) and analyzed using a matched-pairs *t*-test. On average, participants judged that the chatbot would process more sarcasm in the non-literal condition (*M* = −0.03, *SD* = 0.69) than in the literal condition (*M* = −0.87, *SD* = 0.25). This difference, 0.84 BCa 95% CI [0.76, 0.92], was significant, *t*(326) = 20.58, *p* < 0.001, and represented a Cohen’s *d* = 1.14, which is considered as a large effect size (Cohen, 1988).

In a second step, a GLMM was fitted with the same parameters as before. Condition had a significant effect on participants’ *judgements of chatbots’ processing of sarcasm*, *F*(1,1954) = 544.29, *p* < 0.001. With literal privileged information participants were much less likely to select a “Yes” (the chatbot processed the comment as sarcastic) answer than with non-literal privileged information (OR = 0.051, 95% CI [0.040, 0.066], *p* < 0.001). Conversely, this means that a “Yes” answer is 19.61 times more likely to occur in the non-literal condition than in the literal one. A significant random-intercept variance (Var = 1.13, *p* < 0.001) indicated meaningful between-person variability in response levels. To assess the robustness of the effect, a second model was computed that additionally included the continuous control variables (former experience, usage frequency, and their voice-specific counterparts). This did not alter the pattern of results: the effect of condition remained highly significant and identical in magnitude (OR = 0.051, 95% CI [0.039, 0.065], *p* < 0.001). None of the control variables reached significance (all *p* > 0.160), underscoring the robustness of the condition effect against these potential confounds.

These results support our first hypothesis: Privileged information significantly influenced participants’ judgements of chatbots’ processing of sarcasm. Specifically, participants assumed more often that the chatbot would process sarcasm when non-literal privileged information was present, and less often with literal privileged information.

*Prediction of a future action/reaction.* For each scenario participants were asked what they thought the chatbot would answer next. For this they chose between a sarcasm-congruent (=1) and a sarcasm-incongruent (=0) answer. Participants were more likely to anticipate sarcasm-congruent responses following events that implied sarcasm. On average, participants expected such responses in only 3.75% of cases when sarcasm was not suggested, but in 17.7% of cases when sarcasm was suggested. See Table 3 for the distribution across individual scenarios.

**Table 3:** Percentage of answers (rounded) of expected chatbot responses per scenario.

Scenario	Privileged information			
	Non-literal intention		Literal intention	
	Sarcasm-congruent	Sarcasm-incongruent	Sarcasm-congruent	Sarcasm-incongruent
1 Daniel struggles with exam preparations	10	90	3	97
2 Daria curates next semester’s timetable	12	88	4	96
3 Tom registers for his exams	12	88	1	99
4 Franzi wants to join the basketball team	38	62	14	86
5 Ben navigates to the library	15	85	1	99
6 Hannah asks about the canteen’s opening hours	20	80	1	99

A combined sarcasm score was calculated as the mean of all scenarios for each condition. On average, participants expected more sarcasm-congruent answers from chatbots in the non-literal condition (*M* = 0.18, *SD* = 0.31) than in the literal condition (*M* = 0.04, *SD* = 0.12). This difference, −0.14 BCa 95% CI [−0.18, −0.10], was significant, *t*(326) = −7.41, *p* < 0.001, and represented an effect of Cohen’s *d* = 0.41. We interpret this as a small to medium effect size (Cohen, 1988). For the second hypothesis, we fitted a similar GLMM but adapted the model to use a binomial distribution with a logit link function for the dichotomous nature of the outcome. All other model specifications remained unchanged. Condition had a significant effect on the *prediction of a future action/reaction*, *F*(1,1955) = 91.21, *p* < 0.001. With literal privileged information participants were much more likely to select a sarcasm-congruent response than with non-literal privileged information (OR = 7.096, 95% CI [4.745, 10.612], *p* < 0.001). The variance of the random intercept for participants was significant (Var = 1.64, *p* < 0.001), confirming the appropriateness of including participants as a random effect. Including the four control variables (former experience, usage frequency, and their voice-specific counterparts) did not change the pattern of results: the effect of condition remained highly significant and identical in magnitude (OR = 7.200, 95% CI [4.806, 10.787], *p* < 0.001). As none of the control variables reached significance (all *p* > 0.051), the condition effect proved robust to these controls.

This supports our second hypothesis: Privileged information significantly influenced participants’ expectations of a future action or reaction. Specifically, non-literal privileged information increased the likelihood of expecting sarcasm-congruent responses, whereas literal privileged information increased the likelihood of expecting sarcasm-incongruent responses. As previously noted, this conclusion requires caution, as participants formed these judgements after considering the chatbot’s processing.

## Discussion

LLMs and chatbots based on them show a high interaction quality, making our interactions with them feel increasingly natural (Guzman and Lewis, 2020). Are people prone to assume the chatbot would interpret an ambiguous statement in line with their point of view? With this study we explored whether the illusory transparency of intention applies to verbal communication with chatbots.

Let us revisit the scenario where Hannah was asking about the canteen’s opening hours. Depending on whether the chatbot’s answer was helpful or not, the last statement of the scenario could have a literal or non-literal meaning. But do participants falsely expect the chatbot to pick up *conveyed* meaning? In line with our first hypothesis, this proved to be the case. When privileged information was provided that suggested a non-literal, sarcastic meaning, participants significantly more often expected the chatbot to interpret the utterance as such than when privileged information suggested a literal meaning. Not only did they expect the chatbot to detect a non-literal meaning, but in line with hypothesis two they even expected the chatbot’s response to be accordingly. Privileged information suggesting a non-literal, sarcastic meaning led participants to expect a sarcasm-congruent response more often than with privileged information suggesting a literal meaning. In our scenario the response “Sorry for the incorrect information! Let me know if there is anything else I can do to help.” was significantly more often chosen when privileged information suggested that Hannah was not satisfied with the chatbot’s response than “I’m glad I could help you. Enjoy your meal in the cafeteria”!

These results are in line with a strand of research around an egocentric bias in perspective-taking (Epley et al., 2004b; Keysar et al., 1998; Lau et al., 2022), where “one’s prediction of another’s perspective becomes skewed toward one’s own privileged viewpoint.” (Royzman et al., 2003, p. 38). Birch and Bloom (2004) specified that people overestimate what others know when they are knowledgeable, but do not underestimate other’s knowledge when they are ignorant.

Research on spoken communication specifically has shown varying results. Keysar (1994) found no difference between the spoken and written modality (speaking for the illusory transparency of intention), nevertheless the spoken modality was displayed through written scenarios where the text said that the interlocutors spoke to each other. Our results expand these findings by adding actual spoken language. Even with a neutral pronunciation of the ambiguous statements, participants used their knowledge of privileged information to assess the chatbots’ processing. This extends our knowledge of the illusory transparency of intention and the conditions it pertains to.

In contrast to Keysar and Henly (2002), we were able to show that overhearers, in this case our participants who were not involved in the scenario, were affected by privileged information. Whereas Kruger et al. (2005) found no overestimation when participants had to pronounce their e-mails with an opposite phenomenology, we found that with neutral pronunciation the illusory transparency of intention still applied. One difference might be whether participants themselves pronounce the message or whether they listen to it.

In summary our study shows that the illusory transparency of intention applies to verbal communication with LLMs. Participants included privileged information in their assessment of the chatbots’ processing. Our results support the presence of an egocentric bias even in verbal communication.

Furthermore, our findings indicate that the social orientation of humans (Nass et al., 1994) is applicable to illustrations of interactions with LLMs and their voice function. Participants expected chatbots to process ambiguous statements with the same meaning (literal or non-literal) that they gained from the scenarios. Even more so, they expected their answers to be accordingly. Had participants perceived LLMs as mere inanimate objects, it is unlikely that the introduction of privileged information would have yielded an observable effect. Although the current study did not explicitly include a non-agentic control condition, it is reasonable to posit that analogous scenarios involving traditional inanimate objects would not produce similar outcomes. Consequently, these findings underscore a fundamental social dimension within human-AI interactions.

Additionally, our findings have important practical implications. Since LLMs are increasingly integrated into our everyday life, education, and research (Bergmann et al., 2025) it is important to understand how they are perceived and especially which errors can lead to miscommunication. As our experiment showed, people tend to expect chatbots to detect the same meaning they suspected themselves behind a sentence. While LLMs typically respond to context and conversational implicature, they do so without human-like intentional understanding, which can still lead to miscommunication. To account for this, it is essential that users learn to provide background information explicitly. Our findings highlight the necessity of cultivating users’ prompting skills and training them for more successful communication with LLMs.

Finally, we want to emphasize ramifications for higher education. We specifically chose a student sample, as LLMs are already in widespread use among students and young people tend to adapt new technologies faster (Gruenhagen et al., 2024; Shanmugasundaram and Tamilarasu, 2023). We saw that 73% of our sample were frequent or very frequent users of LLMs. This of course changes how students experience education in general and their learning outcomes. Körner et al. (2025) showed that students interacting with a conversational agent who used grounding strategies performed better in a post-study knowledge test and engaged longer with the agent. This could also apply to our results. Students could benefit from chatbots using grounding strategies to prevent false assumptions about the chatbot’s processing. In addition, students should be made aware that even though chatbots and AI tutors appear as social through their messages, they are really only machines.

### Limitations and future research

This study and our results have to be observed under following limitations. By choosing a German student sample, we looked at a group of rather young and experienced users of LLMs. Although the CASA paradigm (Nass et al., 1994) would suggest that inexperienced users would show similar effects, this study might not speak for the whole range of users. For greater generalizability further studies need to consider different age groups, people with less LLM experience, and other nationalities.

Secondly, we collected our data through an online panel and participants were compensated for their participation. This could lead to participants wanting to complete the study as fast as possible and not sensibly go through the scenarios. We chose the Prolific Panel as it has been shown to provide high data quality (Peer et al., 2022). Furthermore, to preclude insensible participation we incorporated attention checks throughout the study and excluded participants who failed them.

The present study did not include a human-human (or other) control condition. Consequently, we can only assume from the literature that the illusory transparency of intention is similar in communication with LLMs and humans, but this equivalence remains an assumption. Further experiments should incorporate appropriate control baselines to empirically test this distinction.

Additionally, it should be mentioned that the order of our survey and repetition of scenarios could have influenced participants’ expectations of chatbots’ responses. In the first round we presented the scenarios and measured participants’ judgements of chatbots’ processing of sarcasm. In the second round, scenarios were presented again and we measured participants’ own perception of sarcasm and asked about the expected responses. It is possible that the two questions about sarcasm perception had an influence on what participants expected the chatbot to answer. We argue that this possible influence is rather small, since for the literal condition only 4% of expected answers were sarcasm-congruent across all scenarios.

We found that participants falsely assess chatbots’ processing and future responses by including privileged information. Future research should investigate whether such errors are detected in communication with LLMs and if so, how they are identified. Moreover, it would be valuable to examine the consequences of these errors for further communication, such as potential reductions in trust or less motivation to use. Another interesting avenue for future research is to examine how preconceived beliefs, particularly assumptions regarding their epistemic status, might influence participants’ judgements of chatbots’ processing.

## Conclusion

Our study investigated whether the illusory transparency of intention applies to scenarios of verbal interactions with LLMs. The findings indicate that perspective-taking in such communication is affected by an egocentric bias, which can lead to miscommunication. This highlights the need to make users aware of cognitive limitations and actively support the development of effective prompting skills. This has particularly significant implications for higher education, as students represent one of the largest user groups and AI is increasingly influencing how they learn.

## Data Availability

The datasets presented in this study can be found in online repositories. The names of the repository/repositories and accession number(s) can be found below: https://osf.io/xcnpa/overview?view_only=9d427ee90d0c4a6cab64d27571008971.

## Footnotes

1
https://osf.io/9sqd5/overview?view_only=81dcb8d920764ba2a44bc68881f9bcf5

2
https://osf.io/xcnpa/overview?view_only=185761c8aeac47cc941a52c2d5559d4b
